# Was China's zero-COVID policy the right choice? The multiple factor analysis of variables that affected the course of COVID-19 pandemic in China

**DOI:** 10.3389/fpubh.2023.1252370

**Published:** 2023-12-06

**Authors:** Marharyta Sobczak, Rafał Pawliczak

**Affiliations:** Department of Immunopathology, Division of Biomedical Science, Faculty of Medicine, Medical University of Lodz, Łódź, Poland

**Keywords:** SARS-CoV-2, COVID-19, China, pandemic, multiple factor analysis

## Abstract

**Background:**

After 3 years of the COVID-19 pandemic and zero-COVID policy, a rapid increase in the number of daily COVID-19 infections was observed in China from November to December 2022. Therefore, we decided to analyze the factors that have been related to the COVID-19 pandemic in China.

**Methods:**

The multiple factor analysis was conducted, using the data from publicly available databases from the beginning of the COVID-19 pandemic to 30 January 2023.

**Results:**

Our study showed that each year of the pandemic in China had different profiles and can be described by different variables: year 2020 was characterized by restrictions, such as international travel controls, stay at home requirements, and health system policies including contact tracing and protection of older adults; year 2021 was characterized by Alpha, Beta, Gamma, and Delta variants; 2022 was characterized by new cases per million, Omicron lineages, and a few restrictions-related variables; and year 2023 was mainly described by the number of new deaths per million and Omicron variant 22B (BA.5) but also by testing and vaccination policies, as well as the number of people fully vaccinated per 100 and total boosters per 100.

**Conclusion:**

The COVID-19 pandemic has changed over time. Therefore, the anti-pandemic policies implemented must be dynamic and adapted to the current situation.

## 1 Introduction

COVID-19 (coronavirus disease 2019) was first reported in China on 31 December 2019. This disease is caused by severe acute respiratory syndrome coronavirus 2 (SARS-CoV-2), which is an enveloped positive-sense RNA virus, that belongs to the family Coronaviridae (1, 2). The infection starts from pneumonia-like symptoms but later leads to lung damage, causing ground-glass opacity lesions (3).

Unfortunately, the SARS-CoV-2 virus is mutating all the time, leading to the origination of the new virus lineages with different genetic variations. Hence, different lineages of virus have different characteristics: some of them may be relatively harmless, and others are more dangerous, as they can spread more easily or be resistant to treatment. Therefore, SARS-CoV-2 lineages are marked as variants under monitoring, variants of interest, and variants of concern (4). On 15 March 2023 World Health Organization (WHO) has updated the definitions of virus variants and the primary actions for particular classification (5). On 30 March 2023, there was one SARS-CoV-2 lineage defined as a variant of interest—XBB.1.5, and there were seven lineages defined as variants under monitoring, namely, BA.2.75, CH.1.1, BQ.1, XBB, XBB.1.16, XBB.1.9.1, and XBF (6).

The start of COVID-19 was the time around the Lunar New Year celebrations in China, which was associated with an increased number of travelers. Therefore, travel restrictions and large social gatherings have been introduced (7). Moreover, through a large number of rigorous public health measures and diagnostic tests, China had almost repressed the domestic transmission of COVID-19 (8). Generally, there were unusually low death counts and effective suppression of COVID-19 spread in South-East and East Asia in comparison to Western countries before the appearance of COVID-19 vaccines. This was explained by the presence of severe acute respiratory syndrome (SARS) and Middle East respiratory syndrome (MERS) epidemics in the past few years. Hence, public health systems in this region were better prepared for similar events (9). However, a rapid increase in daily new COVID-19 cases was observed in China from November to December 2022 (10). Therefore, we decided to analyze the factors that have been related to COVID-19 in China since the beginning of the COVID-19 pandemic.

## 2 Methods

### 2.1 Data search and extraction

For this study, we searched databases such as Oxford COVID-19 Government Response Tracker (11), Our World in Data (12), and CoVariants (13) and collected the data from the beginning of the COVID-19 pandemic to 30 January 2023:

- The number of new cases per million;
- The number of new deaths per million;
- The number of cases with different variants of SARS-CoV-2, which was calculated per million using population number from Our World in Data (12);
- The number of people fully vaccinated per 100;
- The number of total boosters per 100;
- The number of new vaccination 7-day smoothed per million;
- School closing (including universities) on a scale from 0 to 3;
- Workplace closing on a scale from 0 to 3;
- Cancellation of public events on a scale from 0 to 2;
- Restrictions on gathering on a scale from 0 to 4;
- Close public transport on a scale from 0 to 2;
- Stay at home requirements on a scale from 0 to 3;
- Restrictions on internal movement on a scale from 0 to 2;
- International travel controls on a scale from 0 to 4;
- Public information campaigns on a scale from 0 to 2;
- Testing Policy on a scale from 0 to 3;
- Contact tracing on a scale 0 to 2;
- Facial coverings on a scale from 0 to 4;
- Vaccination policy on a scale from 0 to 5;
- Protection of older adults on a scale from 0 to 3.

### 2.2 Statistical analysis

Using collected data, we conducted the multiple factor analysis (MFA), which is similar to principle component analysis (PCA), but used for different types of variables, such as categorical, quantitative, and frequency. MFA is used for the simultaneous exploration of multiway data sets, in which individuals are described by several sets of variables and consist of two steps: At first, it calculates the PCA and normalizes each data table, in which several sets of variables are collected. Moreover, at the second step, these data tables are combined into a joined data table that is analyzed by PCA (14–16). For continuous variables, we calculated the mean score and used standardization and centered around zero, while for categorical variables, we calculated the median. Variables were grouped as new cases, new deaths, SARS-CoV-2 variants, vaccinations, and restrictions, health system policies were grouped as active groups, and years were grouped as supplementary groups. MFA analysis was conducted in R software (version 4.2.2).

## 3 Results

### 3.1 COVID-19 cases and deaths in China from the beginning of the pandemic

**Figure 1:**
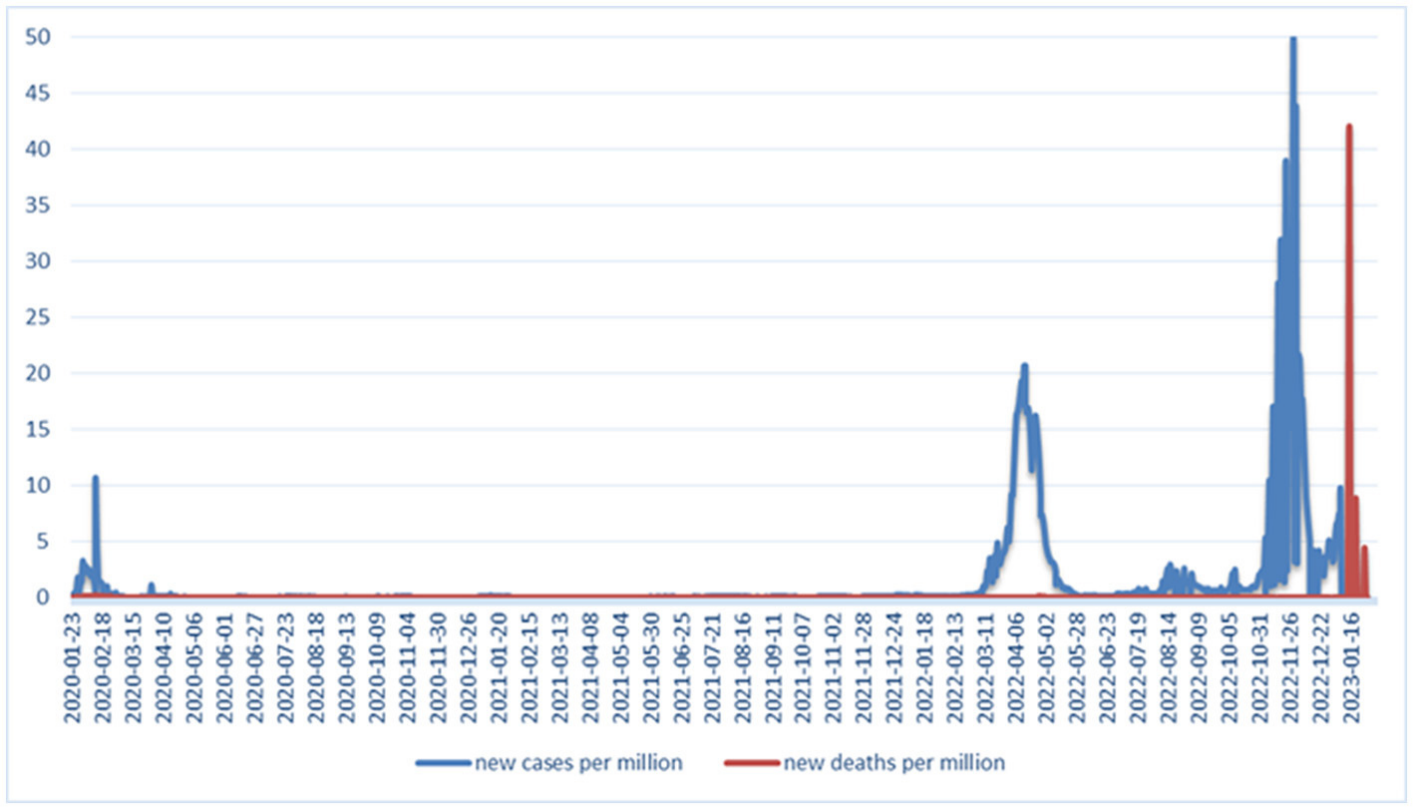
Distribution of COVID-19 cases and deaths in China from the beginning of the pandemic to 30 January 2023.

In the first step, we analyzed the distribution of COVID-19 cases and deaths in China from the beginning of the pandemic (Figure 1). There were three peaks of COVID-19 cases in China during the pandemic: on February 2020, March–May 2022, and October–December 2022, with each peak increasing the number of cases, while there was only one peak of COVID-19 deaths in January 2023.

### 3.2 Contributing variables to the dimension definition

**Figure 2:**
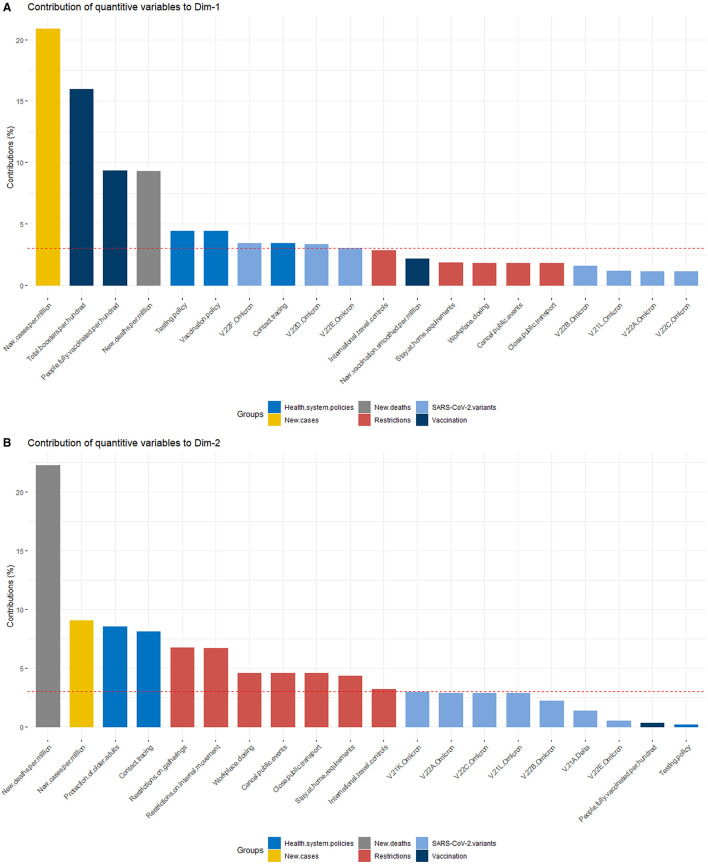
The contribution of quantitative variables to **(A)** Dim-1 and **(B)** Dim-2.

The variable “new cases per million” was the most contributing variable to the definition of dimension 1 (Dim-1), whereas “new deaths per million” was the most contributing variable to the definition of dimension 2 (Dim-2), as shown in Figures 2A, B.

### 3.3 The relationships between analyzed variables

**Figure 3:**
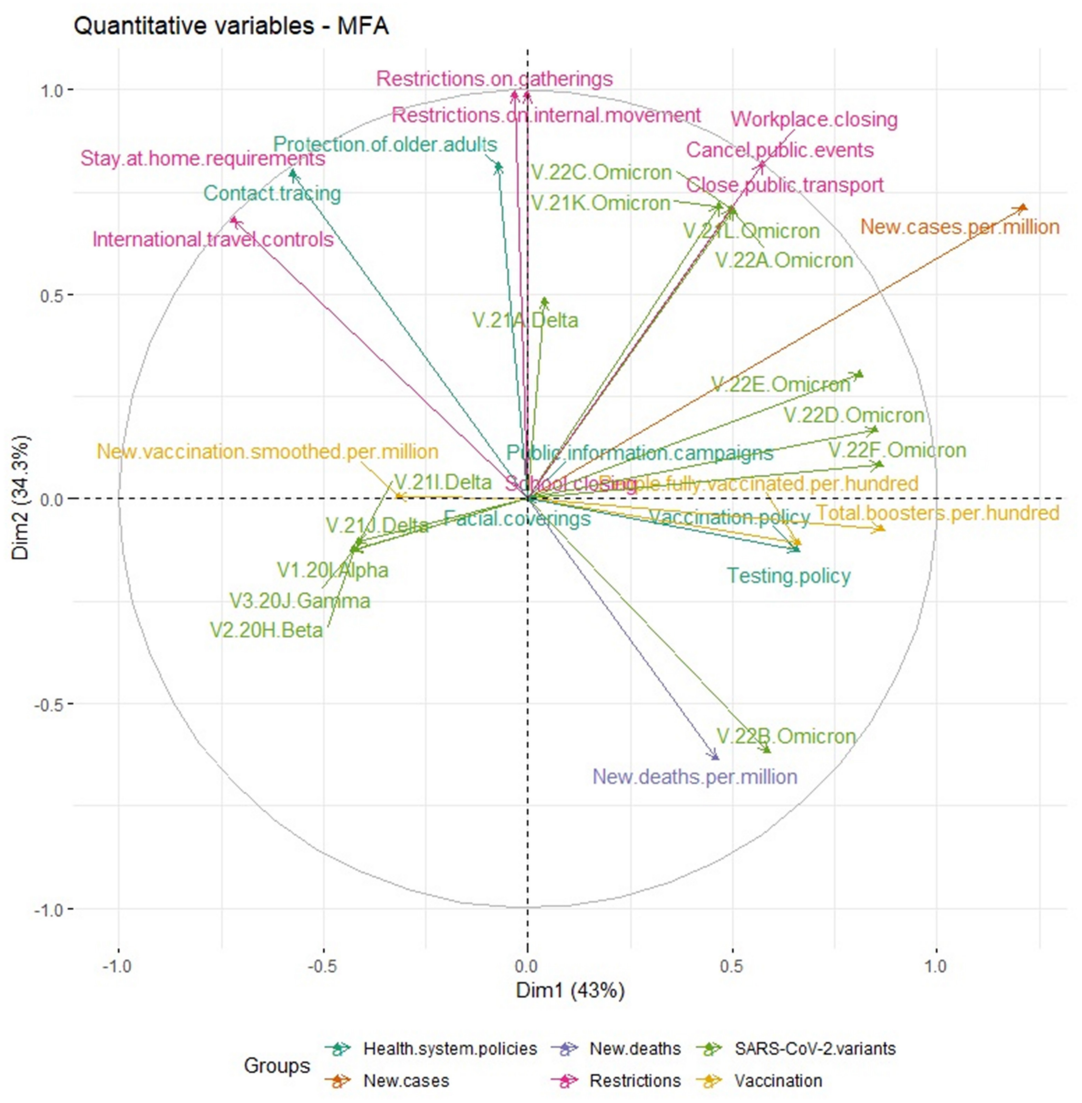
Scatter plot of analyzed variables.

Figure 3 shows a correlation plot of the relationship between analyzed variables. The variable “number of new cases per million” was strongly positively correlated with delta (B.1.617.2) and Omicron SARS-CoV-2 variants, such as 21K (BA.1), 21L (BA.2), 22A (BA.4), 22C (BA.2.12.1), 22D (BA.2.75), 22E (BQ.1), and 22F (XBB) lineages, but also with some variables corresponding to restrictions, health system policies, and number of people fully vaccinated per 100 and total boosters per 100, while “new deaths per million” variable was strongly correlated with Omicron lineage 22B (BA.5) and some variables related to health system policies and number of people fully vaccinated per 100 and total boosters per 100.

### 3.4 The comparison of Chinese profiles in all years of pandemic

**Figure 4:**
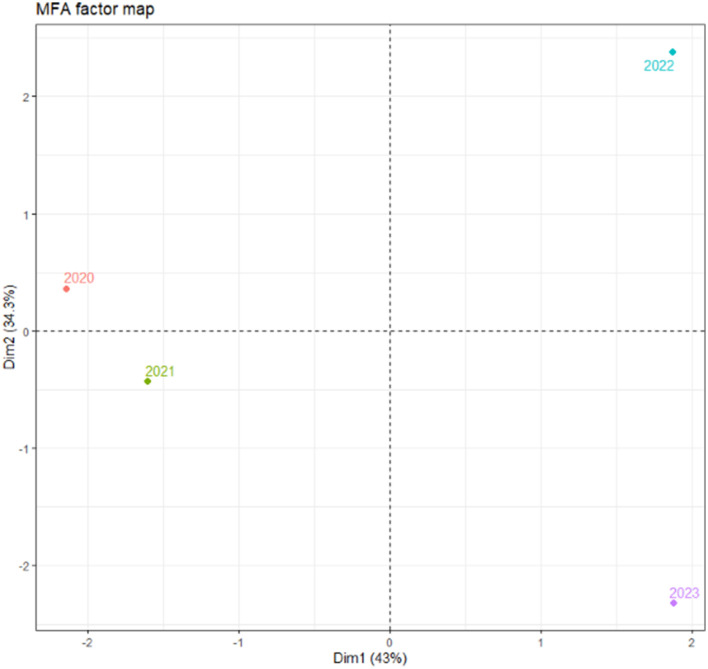
The profiles of China in all years of the COVID-19 pandemic.

In the last step, we compared the profiles of China during the whole period of the COVID-19 pandemic (Figure 4). Each year had different profiles and can be described by different variables: year 2020 was characterized mainly by restrictions, such as international travel controls, stay at home requirements, and also by health system policies including contact tracing and protection of older adults; year 2021 was characterized by the presence of SARS-CoV-2 variants, such as Alpha, Beta, Gamma, and Delta; year 2022 was characterized by new cases per million, Omicron lineages, and few restrictions variables; and January 2023 was predominantly described by the number of new deaths per million and Omicron variant 22B (BA.5) but also by testing and vaccination policies, as well as the number of people fully vaccinated per 100 and total boosters per 100.

## 4 Discussion

In our cross-sectional study, we analyzed the influence of variables pertaining to the number of new COVID-19 cases in China from the beginning of the COVID-19 pandemic. Each year had different profiles and can be described by different variables. Year 2020 was characterized by restrictions, such as international travel controls, stay at home requirements, and health system policies including contact tracing and protection of older adults. Year 2021 was described by SARS-CoV-2 variants, such as Alpha, Beta, Gamma, and Delta, while year 2022 was characterized by new cases per million, Omicron lineages, and few restriction variables. On the other hand, the beginning of 2023 was mainly described by new deaths per million and Omicron variant 22B (BA.5) but also by testing and vaccination policies and the number of people fully vaccinated per 100 and total boosters per 100.

Following the discovery of SARS-CoV2, a novel member of the family Coronaviridae, in early January 2020, China responded with an emergency containment strategy. At the time, the COVID-19 vaccines, treatment, as well as effective operating procedures were unknown. Thus, China, similarly to other countries, focused on the control of viral transmission, monitoring cases of COVID-19, and protection of high-risk groups via NPI (non-pharmaceutical interventions) (17). The crucial factors that affected the effectiveness of NPI measures in China were the rapid introduction of the containment strategy, and sociocultural factors that resulted in social obedience toward governmental policies. Generally, one of the important tools, which used to deter the spread of the virus and identify the contacts between people, is contact tracing. One of the classes of this system based on GPS or location data is the color-based health QR code system which is an innovative system in China. This system classified people through collecting basic information about personal data, travel information, and COVID-19-related information, such as symptoms, vaccination, and results of PCR tests. Then, every individual was classified into a category based on the level of transmission risk using three colors of QR code—green, yellow, and red. Because of privacy data security measures, China introduced some protective measures for privacy in this system (18). Chen et al. (19) in their study claimed that a strategy involving the isolation of infected subjects, disinfection, and health maintenance is crucial for the reduction of the COVID-19 death toll. The above strategy, if implemented properly, was expected to reduce COVID-19-related deaths by 85% in 2020 and by more than 99% in 2022. Moreover, psychosocial problems have become more acute by the outbreak of COVID-19. The study, conducted among Chinese adolescents of 12–18 years, showed that the rates of depression and anxiety symptoms were 43.7% and 37.4%, respectively. Being a senior high school student, residence in Hubei province and female gender were the factors predisposing to developing these symptoms according to multivariable logistic regression analysis (20). However, the prevalence of depression and anxiety symptoms was lower among undergraduates. A higher incidence of developing depression symptoms was observed among the students who were quarantined or isolated while anxiety symptoms were more common among the students whose relatives and friends suffered from COVID-19 (21). Moreover, patients diagnosed with COVID-19 had a higher prevalence of mental problems, insomnia, depression, and anxiety in comparison to non-infected controls (22). Social media exposure was also associated with mental health problems among Chinese citizens (23). Obviously, medical health workers had a higher rate of insomnia, anxiety, depression, somatization, and obsessive-compulsive symptoms when compared with non-medical health workers, as shown in a cross-sectional study conducted in China from 19 February 2020 to March 6, 2020 (24).

In the later stages of the pandemic, a crucial role in restricting the number of COVID-19 cases or deaths is COVID-19 vaccination. According to a survey study conducted among employees of a Chinese geriatric care facility, the respondents possessed were relatively familiar with the topic of COVID-19 vaccines, and the vast majority of them were willing to be vaccinated (25). Medium level of vaccine-related knowledge has been noted among Chinese small-town residents. Moreover, most of the studied population wore masks outside and expressed an optimistic attitude toward the end of the pandemic (26). A survey study conducted on subjects suffering from rheumatoid arthritis confirmed the positive attitude of Chinese society toward the vaccination program, although it pointed out the low availability of vaccine doses as less than half of those willing to participate were vaccinated (27). A cross-sectional study that was conducted on adolescents from three Chinese provinces showed a high level of acceptance rate of COVID-19 vaccination. Only 1.6% of participants have declined the COVID-19 vaccination, mainly due to safety concerns of COVID-19 vaccines (28).

Pan et al. (29) described the contribution of particular SARS-CoV-2 variants to the epidemiological situation in China in 2022. Until November 2022, in Beijing, the dominant variants included Omicron BA.2.2.1, BF.7, and BA.5.2, whereas after mid-November, the dominant variant switched to the clade 22B, predominantly BF.7. Moreover, they observed that the infection peak caused by Omicron sub-variant correlated with the number of flights arrived to Beijing. In January 2022, Omicron sub-variant BA.2 caused COVID-19 cases in Hong Kong. Three doses of BNT162b2 and CoronaVac vaccines have protected against severe and fatal COVID-19 infections (30).

At the end of 2022, there were mass protests in China against zero-COVID policy, according to media news (31–35). The zero-COVID policy has been the foundation of the anti-pandemic strategy in China. However, the lockdowns in the country had negative effects, such as disruption of the economy or food shortage but also difficulties in accessing medical care. Moreover, low morbidity of COVID-19 caused low level of natural immunity in the Chinese population. On the other hand, vaccination hesitancy resulted from no urgent need for vaccination. All of these raise questions about the effectiveness of this policy (36, 37). Because of the spread of the Delta variant, in August 2021, China introduced a novel strategy entitled “Dynamic COVID-zero”, which aimed to minimize the influence of COVID-19 on different socioeconomic areas and balance this with control and prevention of COVID-19 (38). Because of the high infectivity and low pathogenicity of the Omicron variant, China changed its anti-COVID-19 strategy by introducing 30 anti-COVID-19 measures in two waves, the first on 11 November 2022 (20 measures) and the second on 7 December 2022 (10 measures). The above mentioned changes included e.g., alleviation of travel restriction or shortening of isolation period (39). The end of this strategy, which was difficult to maintain against the Omicron variant, was announced on 7 December 2022 by the National Health Commission of China (40). Potentially, a stepwise withdrawal from the zero-COVID strategy could have been achievable as early as April or May 2022 due to warm temperatures, successful vaccination, and well-developed medical infrastructure (41). On 8 January 2023, the zero-COVID policy was stopped in China, which includes missing contact tracing, centralized quarantine, and mass COVID-19 testing (37). As a result, in order to avoid high level of mortality, quick vaccination of older adults might cause a rapid increase in demand for COVID-19 vaccines. Moreover, a sharp increase in the number of COVID-19 infection cases might cause a collapse in hospital infrastructure due to a lack of trained intensive care unit doctors as well as nurses (42).

Unfortunately, our study has some limitations. Not all of the data in the publicly available databases were complete. Moreover, because of the changes in testing strategy, the actual number of reported COVID-19 cases might be different than official data (37). Despite the fact that these limitations may have affected the results of our analysis, in our study, we showed the important factors related to the COVID-19 pandemic in China.

Although most of the countries adopted similar anti-COVID-19 prevention measures, they had to adapt their strategies according to their individual needs, e.g., economic or healthcare status. European Union (EU) members presented a different attitude toward the COVID-19 pandemic, especially when it came to the general lack of control of cross-border movements between the states and the choice of daily life restrictions and their severity. Moreover, during the COVID-19 pandemic, the crucial factors that affected the number of COVID-19 deaths in EU were the socioeconomic status and the availability of specialized equipment in the medical care facilities (43). Noteworthy, the measures implemented in Europe, similar to the Chinese strategy, have proven to be relatively successful in limiting SARS-CoV-2 spread. Our study pointed out the main factors that influenced the number of COVID-19 cases in the course of the pandemic in China starting from the year 2020 to the beginning of 2023. At the very beginning of the pandemic, the crucial role was played by anti-COVID-19 countermeasures of China, while in the latter stages, the number of cases was affected mostly by the vaccination status and the presence of COVID-19 variants.

## Data availability statement

Publicly available datasets were analyzed in this study. This data can be found here: https://ourworldindata.org; https://www.eurocontrol.int; https://www.bsg.ox.ac.uk/.

## Author contributions

MS searched the databases, prepared the analysis, interpreted the results, and wrote the manuscript. RP supervised the overall study, analyzed the data, and critically reviewed the manuscript. All authors have read and approved the final manuscript.

## References

[1] Zhan Y Li XP Yin JY. COVID-19 one year later: a retrospect of CRISPR-Cas system in combating COVID-19. Int J Biol Sci. (2021) 17:2080–8. 10.7150/ijbs.60655 34131407 PMC8193275

[2] Umakanthan S Sahu P Ranade AV Bukelo MM Rao JS Abrahao-Machado LF. Origin, transmission, diagnosis and management of coronavirus disease 2019 (COVID-19). Postgrad Med J. (2020) 96(1142):753−8. 10.1136/postgradmedj-2020-138234 32563999 PMC10016932

[3] Han Z Battaglia F Terlecky SR. Discharged COVID-19 patients testing positive again for SARS-CoV-2 RNA: A minireview of published studies from China. J Med Virol. (2021) 93:262–74. 10.1002/jmv.26250 32609390 PMC7361580

[4] Health C for D R. SARS-CoV-2 Viral Mutations: Impact on COVID-19 Tests. FDA. (2023). Available online at: https://www.fda.gov/medical-devices/coronavirus-covid-19-and-medical-devices/SARS-CoV-2-viral-mutations-impact-covid-19-tests (accessed April 14, 2023).

[5] Updated Working Definitions Primary Actions for SARSCoV2 Variants. (2023). Available online at: https://www.who.int/publications/m/item/updated-working-definitions-and-primary-actions-for–SARS-CoV-2-variants (accessed April 16, 2023).

[6] Tracking SARS-CoV-2 Variants (2023). Available online at: https://www.who.int/activities/tracking-SARS-CoV-2-variants (accessed April 16, 2023).

[7] Read JM Bridgen JRE Cummings DAT Ho A Jewell CP. Novel coronavirus 2019-nCoV (COVID-19): early estimation of epidemiological parameters and epidemic size estimates. Philos Trans R Soc Lond B Biol Sci. 376:20200265. 10.1098/rstb.2020.0265 34053269 PMC8165596

[8] Tian D Song Y Zhang M Pan Y Ge Z Zhang Y. Genomic, immunological, and clinical analysis of COVID-19 vaccine breakthrough infections in Beijing, China. J Med Virol. (2022) 94:2237–49. 10.1002/jmv.27636 35112366 PMC9015436

[9] Lee B Ibrahim SA Zhang T. Mobile apps leveraged in the COVID-19 pandemic in east and south-east asia: review and content analysis. JMIR Mhealth Uhealth. (2021) 9:e32093. 10.2196/32093 34748515 PMC8589041

[10] China COVID - Coronavirus Statistics - Worldometer (2023). Available online at: https://www.worldometers.info/coronavirus/country/china/ (accessed April 15, 2023).

[11] COVID-19 Government Response Tracker (2023). Available online at: https://www.bsg.ox.ac.uk/research/covid-19-government-response-tracker (accessed February 7, 2022).

[12] Our World in Data. Our World in Data. (2023). Available online at: https://ourworldindata.org (accessed February 7, 2022).

[13] CoVariants. (2023). Available online at: https://covariants.org/ (accessed February 7, 2022).

[14] Abdi H Williams LJ Valentin D. Multiple factor analysis: principal component analysis for multitable and multiblock data sets: multiple factor analysis. WIREs Comp Stat. (2013) 5:149–79. 10.1002/wics.1246

[15] Bécue-Bertaut M Pagès J. Multiple factor analysis and clustering of a mixture of quantitative, categorical and frequency data. Comput Stat Data Anal. (2008) 52:3255–68. 10.1016/j.csda.2007.09.023

[16] de Tayrac M Lê S Aubry M Mosser J Husson F. Simultaneous analysis of distinct Omics data sets with integration of biological knowledge: multiple factor analysis approach. BMC Genomics. (2009) 10:32. 10.1186/1471-2164-10-32 19154582 PMC2636827

[17] Zhou L Wu Z Li Z Zhang Y McGoogan JM Li Q. 100 days of COVID-19 prevention and control in China. Clin Infect Dis. (2020) 5:ciaa725. 10.1093/cid/ciaa725

[18] Cheng ZJ Zhan Z Xue M Zheng P Lyu J Ma J. Public health measures and the control of COVID-19 in China. Clin Rev Allergy Immunol. (2023) 64:1–16. 10.1007/s12016-021-08900-2 34536214 PMC8449219

[19] Chen JM Li GH Ji YF Sun MH Gong HY Chen RX. A highly powerful nonspecific strategy to reduce COVID-19 deaths. J Med Virol. (2022) 94:5051–5. 10.1002/jmv.27949 35729074 PMC9349517

[20] Zhou SJ Zhang LG Wang LL Guo ZC Wang JQ Chen JC. Prevalence and socio-demographic correlates of psychological health problems in Chinese adolescents during the outbreak of COVID-19. Eur Child Adolesc Psychiatry. (2020) 29:749–58. 10.1007/s00787-020-01541-4 32363492 PMC7196181

[21] Huang Y Su X Si M Xiao W Wang H Wang W. The impacts of coping style and perceived social support on the mental health of undergraduate students during the early phases of the COVID-19 pandemic in China: a multicenter survey. BMC Psychiatry. (2021) 21:530. 10.1186/s12888-021-03546-y 34706690 PMC8549419

[22] Lu X Xie Y Feng H Liu Z Ouyang K Hou B. Psychological impact on COVID-19 patients during the outbreak in China: a case-control study. Psychiatry Res. (2021) 305:114180. 10.1016/j.psychres.2021.114180 34461357 PMC8381686

[23] Gao J Zheng P Jia Y Chen H Mao Y Chen S. Mental health problems and social media exposure during COVID-19 outbreak. PLoS ONE. (2020) 15:e0231924. 10.1371/journal.pone.0231924 32298385 PMC7162477

[24] Zhang W Wang K Yin L Zhao W Xue Q Peng M. Mental health and psychosocial problems of medical health workers during the COVID-19 epidemic in China. Psychother Psychosom. (2020) 9:1–9. 10.1159/000507639 32272480 PMC7206349

[25] Li H Cheng L Tao J Chen D Zeng C. Knowledge and willingness to receive a COVID-19 vaccine: a survey from Anhui Province, China. Hum Vaccin Immunother. 18:2024064. 10.1080/21645515.2021.2024064 35130110 PMC8993089

[26] Yu SY Luo JJ Cui HY Shan KS Xu L Ding L. Knowledge, Attitudes, and practices toward COVID-19 and vaccines among Chinese small-town residents: a cross-sectional study. Am J Trop Med Hyg. (2022) 107:551–6. 10.4269/ajtmh.22-0031 35895333 PMC9490646

[27] Yi Z Yao Z Xu D Xu C Fang W Guo Z. Attitudes toward COVID-19 vaccination: a survey of Chinese patients with rheumatic diseases. Vaccines (Basel). (2022) 10:1604. 10.3390/vaccines10101604 36298469 PMC9611697

[28] Li T Qi R Chen B Luo Y Zhang W Zhou YH. COVID-19 vaccination coverage among adolescents aged 12–17 years in three provinces of eastern China: a cross-sectional survey, 2021. Front Public Health. (2022) 10:919190. 10.3389/fpubh.2022.919190 35937249 PMC9355633

[29] Pan Y Wang L Feng Z Xu H Li F Shen Y. Characterisation of SARS-CoV-2 variants in Beijing during 2022: an epidemiological and phylogenetic analysis. Lancet. (2023) 401:664–72. 10.1016/S0140-6736(23)00129-0 36773619 PMC9949854

[30] McMenamin ME Nealon J Lin Y Wong JY Cheung JK Lau EHY. Vaccine effectiveness of one, two, and three doses of BNT162b2 and CoronaVac against COVID-19 in Hong Kong: a population-based observational study. Lancet Infect Dis. (2022) 22:1435–43. 10.1016/S1473-3099(22)00345-0 35850128 PMC9286709

[31] Time. Xi's ‘Trap': Why China Can't Just End Its Zero-COVID Policy. (2022). Available online at: https://time.com/6237990/china-end-zero-covid-policy/ (accessed April 19, 2023).

[32] Davidson H Yu V. Clashes in Shanghai as protests over zero-Covid policy grip China. In: The Guardian. (2022). Available online at: https://www.theguardian.com/world/2022/nov/28/clashes-in-shanghai-as-protests-over-zero-covid-policy-grip-china (accessed April 19, 2023).

[33] Council on Foreign Relations,. Did China's Street Protests End Harsh COVID Policies? (2022). Available online at: https://www.cfr.org/blog/did-chinas-street-protests-end-harsh-covid-policies (accessed April 19, 2023).

[34] The Real Importance of China's ‘Zero COVID' Protests. (2022). Available online at: https://thediplomat.com/2022/11/the-real-importance-of-chinas-zero-covid-protests/ (accessed April 19, 2023).

[35] Zero-Covid anger grows after deadly China fire,. BBC News. (2022). Available online at: https://www.bbc.com/news/av/world-asia-china-63770974 (accessed April 19, 2023).

[36] Yuan S. Zero COVID in China: what next? Lancet. (2022). 399:1856–7. 10.1016/S0140-6736(22)00873-X 35569453 PMC9098203

[37] The Lancet Regional Health – Western Pacific. The end of zero-COVID-19 policy is not the end of COVID-19 for China. Lancet Reg Health West Pac. (2023) 30:100702. 10.1016/j.lanwpc.2023.100702 36741798 PMC9887076

[38] Liu J Liu M Liang W. The dynamic COVID-zero strategy in China. China CDC Wkly. (2022) 4:74–5. 10.46234/ccdcw2022.015 35186372 PMC8837441

[39] Tang S Wang X Tang B He S Yan D Huang C. Threshold conditions for curbing COVID-19 with a dynamic zero-case policy derived from 101 outbreaks in China. BMC Public Health. (2023) 23:1084. 10.1186/s12889-023-16009-8 37280554 PMC10242611

[40] Burki T. Moving away from zero COVID in China. Lancet Respir Med. (2023) 11:132. 10.1016/S2213-2600(22)00508-2 36535298 PMC9757876

[41] Chen JM Chen YQ. China can prepare to end its zero-COVID policy. Nat Med. (2022) 28:1104–5. 10.1038/s41591-022-01794-3 35383312

[42] Wilson O Flahault A. China's U-turn in its COVID-19 policy. Anaesth Crit Care Pain Med. (2023) 42:101197. 10.1016/j.accpm.2023.101197 36646356 PMC9837380

[43] Bourdin S Ben Miled S Salhi J. The Drivers of Policies to Limit the Spread of COVID-19 in Europe. J Risk Financ Manage. (2022) 15:67. 10.3390/jrfm15020067

